# Sequential immunization with SARS-CoV-2 RBD vaccine induces potent and broad neutralization against variants in mice

**DOI:** 10.1186/s12985-021-01737-3

**Published:** 2022-01-04

**Authors:** Shuo Song, Bing Zhou, Lin Cheng, Weilong Liu, Qing Fan, Xiangyang Ge, Hua Peng, Yang-Xin Fu, Bin Ju, Zheng Zhang

**Affiliations:** 1 Institute for Hepatology, National Clinical Research Center for Infectious Disease, Shenzhen Third People’s Hospital, Shenzhen, Guangdong Province China; 2 The Second Affiliated Hospital, School of Medicine, Southern University of Science and Technology, Shenzhen, Guangdong Province China; 3 Shenzhen Research Center for Communicable Disease Diagnosis and Treatment of Chinese Academy of Medical Science, Shenzhen, Guangdong Province China; 4 Guangdong Key Laboratory for Anti-Infection Drug Quality Evaluation, Shenzhen, Guangdong Province China; 5 Institute of Biophysics, Chinese Academy of Sciences, Beijing, China; 6 Guangzhou Laboratory, and Bioland Laboratory (Guangzhou Regenerative Medicine and Health Guangdong Laboratory), Guangzhou, China; 7 Department of Basic Medical Sciences, School of Medicine, Tsinghua University, Beijing, China

**Keywords:** SARS-CoV-2 variant, Inactivated vaccine, Mutant RBD vaccine, Sequential immunization, Broadly neutralizing antibody

## Abstract

The current COVID-19 pandemic caused by constantly emerging SARS-CoV-2 variants still poses a threat to public health worldwide. Effective next-generation vaccines and optimized booster vaccination strategies are urgently needed. Here, we sequentially immunized mice with a SARS-CoV-2 wild-type inactivated vaccine and a heterologous mutant RBD vaccine, and then evaluated their neutralizing antibody responses against variants including Beta, Delta, Alpha, Iota, Kappa, and A.23.1. These data showed that a third booster dose of heterologous RBD vaccine especially after two doses of inactivated vaccines significantly enhanced the GMTs of nAbs against all SARS-CoV-2 variants we tested. In addition, the WT and variants all displayed good cross-immunogenicity and might be applied in the design of booster vaccines to induce broadly neutralizing antibodies.

**Supplementary Information:**

The online version contains supplementary material available at 10.1186/s12985-021-01737-3.

## Introduction

The coronavirus disease 2019 (COVID-19) pandemic caused by severe acute respiratory syndrome coronavirus 2 (SARS-CoV-2) has led to more than 200 million infections and 4 million deaths worldwide since the outbreak began. To date, approximately 18 kinds of SARS-CoV-2 vaccines, including inactivated, mRNA, DNA, adenovirus vector, and recombinant subunit vaccines, have been developed to control the pandemic, some of which have received a wide range of approvals for use [1]. However, with the emergence of various SARS-CoV-2 variants, the protection afforded by current vaccines has decreased significantly, which has been proven for several waves of the COVID-19 pandemic caused by Alpha, Beta, Iota, and Kappa variants. Especially, the Delta variant has spread rapidly to many countries and become the predominant variant worldwide. These SARS-CoV-2 variants continuously threaten global public health. Therefore, it is critical to develop more efficient vaccines and immunization strategies for controlling the COVID-19 pandemic and preventing breakthrough infection of variants after vaccination.

A third booster dose based on conventional two-dose vaccination is usually considered as a direct and effective strategy to enhance the immune responses such as the titer of neutralizing antibodies (nAbs). Recently, clinical data have shown that a third dose of homologous mRNA vaccine could substantially reduce the new viral infection and the risk of severe illness [2]. As compared with homologous boosters, the heterologous prime-boost strategy may improve the levels of nAbs more effectively [3]. However, it is unclear whether the sequential immunization with vaccines based on different platforms could increase the antibody response to SARS-CoV-2 variants. Two recent studies have made important strides toward enhancing broadly neutralizing activities against SARS-CoV-2 variants by heterologous ChadOx1/BNT162b2 vaccination, a combination of adenovirus-vectored vaccine and mRNA vaccine [4, 5]. However, the immunogenicity and efficacy of other vaccine combinations have not been comprehensively analyzed, especially using the SARS-CoV-2 variant-based vaccine. Two doses of inactivated vaccines are widely used vaccines but which type of booster vaccines have not been reported.

In this study, we sequential immunized mice with a wild-type (WT) inactivated vaccine and a mutant receptor-binding domain (RBD) vaccine, and evaluated the neutralizing antibody response to SARS-CoV-2 WT strain or variants including Beta, Delta, Alpha, Iota, Kappa, or A.23.1, which will offer more options choices in vaccination strategies to control the current COVID-19 pandemic.

## Materials and methods

### Animals, immunization and serum sample collection

To assess the immunogenicity of the SARS-CoV-2 vaccines, 80 specific pathogen-free (SPF) female BALB/c mice were randomly divided into 8 groups (n = 10) and then immunized intraperitoneally with the SARS-CoV-2 inactivated vaccine or RBD subunit vaccines at an interval of 2 weeks in different procedures or immunized with aluminum adjuvant, which served as a control. No adverse events were observed throughout the experiment. The serum samples were collected 14 days after the last dose and heat inactivated at 56 °C for 30 min before use.

### SARS-CoV-2 pseudovirus-based neutralization assay

SARS-CoV-2 pseudovirus was generated by cotransfection of HEK-293 T cells with a SARS-CoV-2 spike-expressing plasmid and an env-deficient HIV-1 backbone vector (pNL4-3.Luc.R-E-). Two days post-transfection, the culture supernatant was harvested, clarified by centrifugation, filtered and stored at − 80 °C. To determine the neutralizing activity, serially diluted serum samples were incubated with an equal volume of SARS-CoV-2 pseudovirus at 37 °C for 1 h. HEK-293 T-hACE2 cells were subsequently added to the plates. After a 48 h incubation, the culture medium was removed, and 100 μL of Bright-Lite Luciferase reagent (Vazyme Biotech) was added to the cells. After a 2-min incubation at RT, 90 μL of cell lysate was transferred to 96-well white solid plates to measure luminescence using a Varioskan™ LUX multimode microplate reader (Thermo Fisher Scientific). The 50% inhibitory dilution (ID_50_) was calculated using GraphPad Prism 8.0 software by the log (inhibitor) versus normalized response—variable slope (four parameters) model.

## Main text

**Fig. 1:**
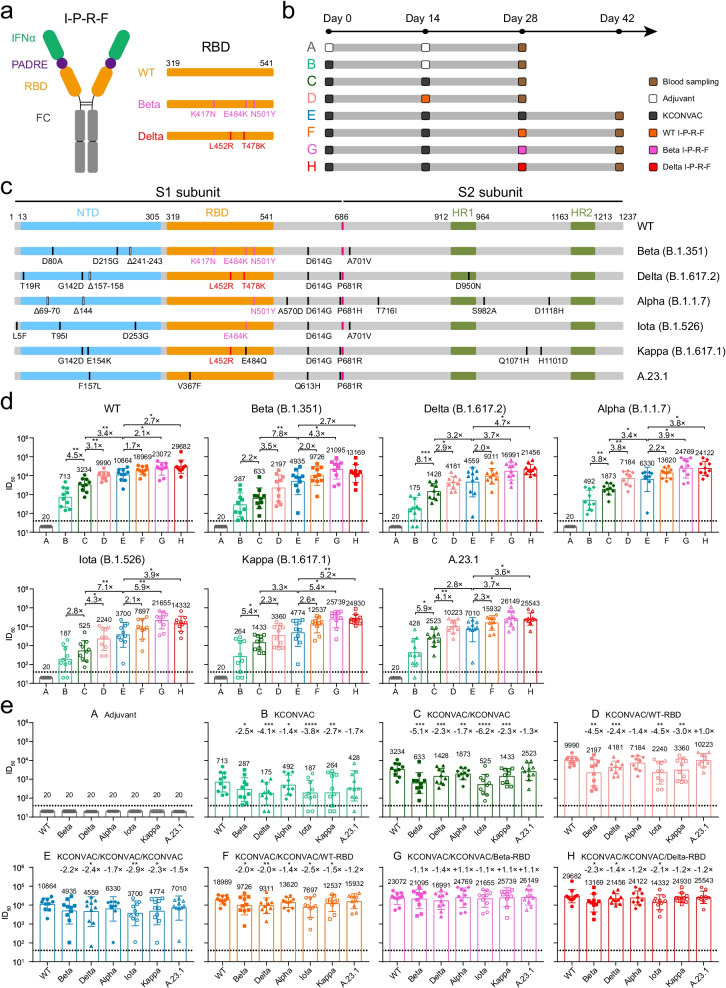
Potent and broad neutralization against SARS-CoV-2 variants elicited by sequential immunization with a wild-type inactivated vaccine and a mutant RBD subunit vaccine in mice. **a** Schematic diagram of the RBD dimer vaccine named I-P-R-F [6]. The structural elements show IFNα, Pan-DR epitope (PADRE), RBD, and immunoglobulin Fc fragment. The RBD regions of three kinds of I-P-R-F, including WT and Beta and Delta variants, are shown on the right, and mutations are labeled as sticks. **b** Schematic representation of the immunization schedule and blood sample collection of mice in 8 groups (Groups A-H, n = 10 in each group). **c** Schematic representation of mutations located in viral spike proteins of the Beta, Delta, Alpha, Iota, Kappa, and A.23.1 variants. **d** Neutralization titers against SARS-CoV-2 WT and variants of vaccine-elicited mouse serum. The data are shown for different pseudoviruses. **e** Neutralization titers against SARS-CoV-2 WT and variants of vaccine-elicited mouse serum. The data are shown for different groups and all comparisons are made to the WT readings. Geometric mean titers, in ID_50_ values, were calculated and are written in each column. The error bars are standard deviations, and the symbols represent individual mice. The dotted line indicates the limit of detection (1:40 dilution) for the assay. Non-neutralizing serum was assigned a value of one-half of the limit of detection for visualization (1:20 dilution). Statistical analysis was performed with paired or unpaired *t* tests using GraphPad Prism 8.0 software. *P < 0.05; **P < 0.01; ***P < 0.001; ****P < 0.0001

Previously, Sun et al. constructed an interferon-armed RBD dimer vaccine inducing the production of high titers of long-lasting nAbs in mice and rhesus macaques [6]. Here, we acquired three kinds of RBD dimer vaccines carrying WT, Beta, and Delta RBD proteins from the Peng group (Fig. 1a) and obtained a WT inactivated vaccine from Shenzhen Kangtai Biological Products Co., Ltd. [7]. To be paralleled with the clinical trials, we immunized mice with a human IFN-Pan-RBD-human Fc containing one mutation in IFN for cross-binding to the mouse IFN receptor (named I-P-R-F, or V-01). Although V-01 binds to the mouse IFN receptor with very low affinity and mediates weaker immune response than V-01 in human, we still observed significant booster effect of V-01 for the inactivated vaccine. As shown in the schematic (Fig. 1b), 80 female mice were randomly divided into 8 groups. In Group A, 10 mice received two doses of aluminum adjuvant and served as a negative control. In Groups B, C, and E, 30 mice were injected with one, two, or three doses of 1 μg of the inactivated vaccine, respectively. The rest of the mice were sequentially immunized with same doses of 1 μg of the inactivated vaccine and RBD vaccines: one dose of inactivated vaccine and a WT RBD booster in Group D and two doses of inactivated vaccine followed by a WT, Beta, or Delta RBD booster in Groups F, G, and H, respectively.

Based on the HIV-1 backbone, we constructed a series of SARS-CoV-2 pseudoviruses bearing spike proteins of the WT strain or Beta, Delta, Alpha, Iota, Kappa, or A.23.1 variant (Fig. 1c). A substitution at the E484 residue was found in the RBD region of the Beta, Iota, and Kappa variants. The Beta and Alpha variants shared a common mutation of N501Y. Both the Delta and Kappa variants carried the L452R mutation. This phenomenon of sharing common mutations suggests that viral infection or vaccination might induce cross neutralization against SARS-CoV-2 variants. Two weeks after the last vaccination, all serum samples were collected and then tested by a pseudovirus neutralizing assay (Fig. 1d, Additional file 1: Fig. S1, and Additional file 1: Table S1). In Groups B, C, and E, the geometric mean titers (GMTs) of nAbs in vaccinated mice gradually increased with the increase in the number of immunizations.

It is widely known that SARS-CoV-2 uses its RBD to recognize the host cell receptor (angiotensin-converting enzyme 2, ACE2). Therefore, the RBD should be a good immunogen and could trigger an effective antibody response [8, 9]. Compared with the homologous booster, sequential immunization with inactivated vaccine and RBD vaccine indeed induced more potent neutralization against SARS-CoV-2 WT and mutant pseudoviruses (Group D vs. Group C, Group F vs. Group E). Considering that the Beta variant exhibited the greatest reduction in neutralizing activity thus far and that the Delta variant dominated the current wave of the COVID-19 pandemic worldwide, we also evaluated the neutralizing antibody responses elicited by a third booster of the Beta-RBD vaccine or Delta-RBD vaccine. As shown in Fig. 1d, the GMTs of serum nAbs against WT SARS-CoV-2 and variants in Group G or Group H were the highest among all immunization strategies, suggesting that mutant RBD vaccines following two doses of inactivated vaccines could induce more broadly neutralizing antibody responses against variants.

To further analyze the spectrum of serum nAbs elicited by all of the aforementioned immunization strategies, we adjusted these neutralization results by different groups to compare their neutralizing activities against SARS-CoV-2 variants. As shown in Fig. 1e, serum nAb levels of mice that received either one or two doses of vaccines (Groups B, C, and D) significantly declined against variants including Beta, Delta, Iota, and Kappa. Similar to previous reports [10, 11], the L452R and E484K/Q mutations in the RBD contributed to the significant decline in neutralizing activities. In contrast, the Alpha or A.23.1 variant carrying a single mutation (N501Y or V367F, respectively) in the RBD slightly escaped the neutralization of vaccine-induced serum, which was also consistent with other studies [12, 13]. Encouragingly, the serum of mice that received three doses of vaccines (Groups E, F, G, and H) maintained quite a high level of nAbs against all tested SARS-CoV-2 variants, with degrees of neutralization that declined less than threefold compared to that against WT. Notably, two doses of WT inactivated vaccines followed by a third booster of Beta-RBD vaccine elicited the most broadly neutralizing antibody response in mice of Group G. All SARS-CoV-2 variants we tested did not escape from the neutralization of serum elicited by inactivated/inactivated/Beta-RBD vaccines. Overall, in this study, although the viral clearance from various organs, histopathology studies, and T-cell responses are not investigated due to the limitation of live virus and animal model, the data based on the neutralizing antibody responses are still certainly to prove that either WT or mutant RBD might be a suitable candidate for the design of a next-generation broad vaccine. There may be no need to generate each SARS-CoV-2 variant, which will save resources and time that require to develop each variants.

## Conclusion

In conclusion, our results demonstrated that sequential immunization with the SARS-CoV-2 inactivated vaccine and RBD subunit vaccine indeed induced potent and broad neutralization against variants. Currently, a large number of people around the world have received two doses of inactivated vaccines but are still at the risk of breakthrough infections by various SARS-CoV-2 variants. Based on our data in mice, a third booster of a RBD vaccine following two doses of widely used WT inactivated vaccines should be a good choice and an effective strategy to combat SARS-CoV-2 variants and control the current COVID-19 pandemic.

## Supplementary Information

**Additional file 1. Figure S1**. Neutralization curves against SARS-CoV-2 WT and variants of vaccine-elicited mice serum and a positive control mAb. All serum samples were serially 3-fold diluted from 1:40. A positive control mAb (P2C-1F11) was serially 3-fold diluted from 5 μg/ml. A 50% reduction in viral infectivity was indicated by a horizontal dashed line.** Table S1**. The geometric mean titers of vaccine-elicited mice serum against SARS-CoV-2 WT and variants.

## Data Availability

We are happy to share reagents and information in this study upon request.

## Abbreviations

SARS-CoV-2: Severe Acute Respiratory Syndrome Coronavirus 2
nAb: Neutralizing antibody
ACE2: Angiotensin-converting enzyme 2
RBD: Receptor binding domain
WT: Wild type
