# Supplementary material for: Sequential immunization with SARS-CoV-2 RBD vaccine induces potent and broad neutralization against variants in mice
Source: Virol J. 2022 Jan 4;19:2. doi: 10.1186/s12985-021-01737-3 (PMC8724645; doi:10.1186/s12985-021-01737-3)
Supplement: Supplementary file 1 — Additional file 1. Figure S1. Neutralization curves against SARS-CoV-2 WT and variants of vaccine-elicited mice serum and a positive control mAb. All serum samples were serially 3-fold diluted from 1:40. A positive control mAb (P2C-1F11) was serially 3-fold diluted from 5 μg/ml. A 50% reduction in viral infectivity was indicated by a horizontal dashed line. Table S1. The geometric mean titers of vaccine-elicited mice serum against SARS-CoV-2 WT and variants. [file 12985_2021_1737_MOESM1_ESM.docx]

**Supplementary Information**

**
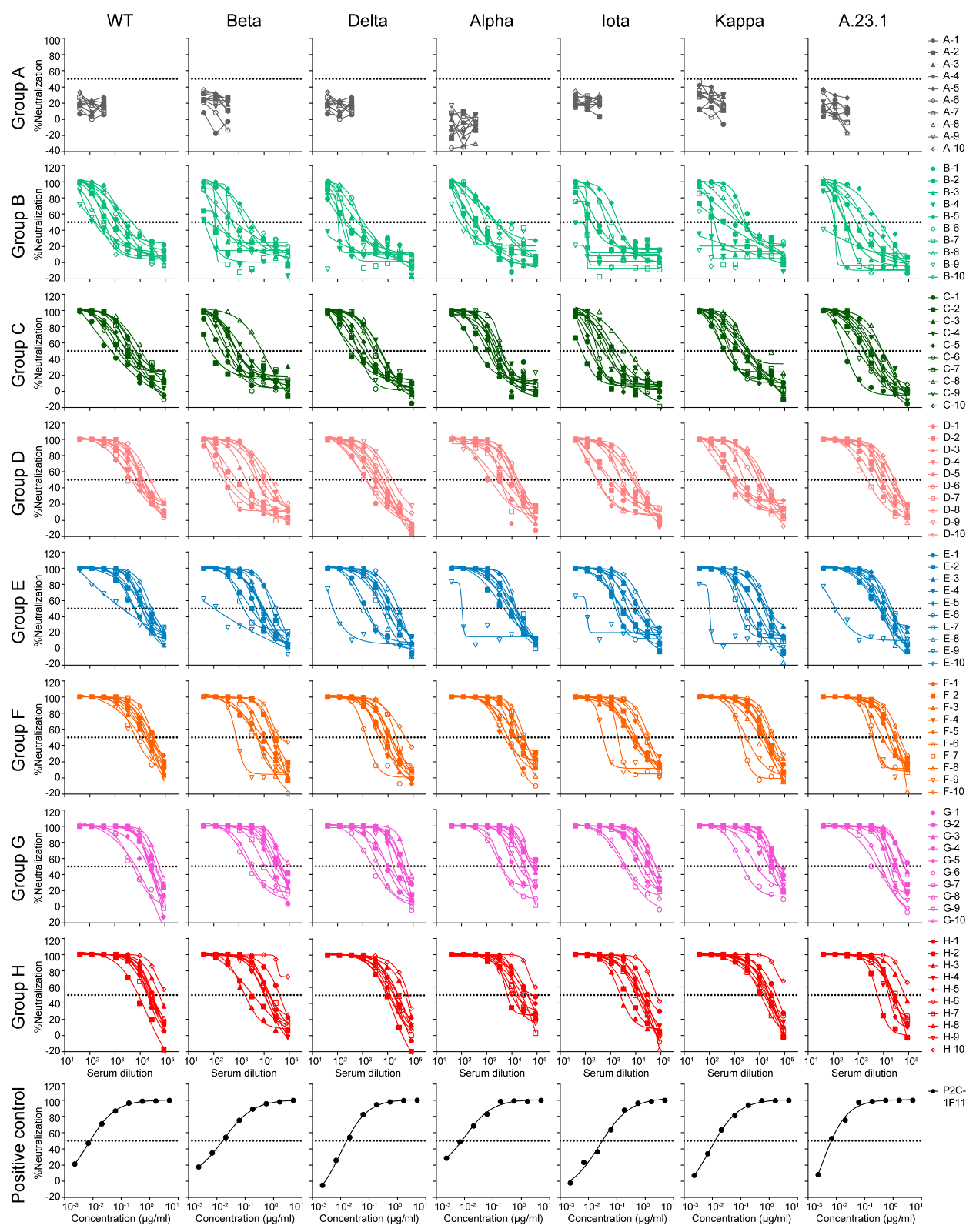
**

**Supplementary Figure S1. Neutralization curves against SARS-CoV-2 WT and variants of vaccine-elicited mice serum and a positive control mAb.**

All serum samples were serially 3-fold diluted from 1:40. A positive control mAb (P2C-1F11) was serially 3-fold diluted from 5 μg/ml. A 50% reduction in viral infectivity was indicated by a horizontal dashed line.

**Supplementary Table S1. The geometric mean titers of vaccine-elicited mice serum against SARS-CoV-2 WT and variants.**

| **Mice** | **Pseudoviruses** | | | | | | |  | **Mice** | **Pseudoviruses** | | | | | | |
| --- | --- | --- | --- | --- | --- | --- | --- | --- | --- | --- | --- | --- | --- | --- | --- | --- |
|  | **WT** | **Beta** | **Delta** | **Alpha** | **Iota** | **Kappa** | **A.23.1** |  |  | **WT** | **Beta** | **Delta** | **Alpha** | **Iota** | **Kappa** | **A.23.1** |
| **A-1** | 20 | 20 | 20 | 20 | 20 | 20 | 20 |  | **E-1** | 5673 | 4706 | 1419 | 9093 | 1589 | 3171 | 7338 |
| **A-2** | 20 | 20 | 20 | 20 | 20 | 20 | 20 |  | **E-2** | 19576 | 3045 | 9249 | 4802 | 3143 | 7323 | 6590 |
| **A-3** | 20 | 20 | 20 | 20 | 20 | 20 | 20 |  | **E-3** | 20627 | 8309 | 17294 | 7125 | 14473 | 20554 | 18377 |
| **A-4** | 20 | 20 | 20 | 20 | 20 | 20 | 20 |  | **E-4** | 11677 | 9315 | 12916 | 8739 | 8568 | 16510 | 9245 |
| **A-5** | 20 | 20 | 20 | 20 | 20 | 20 | 20 |  | **E-5** | 14452 | 20593 | 15784 | 13024 | 11253 | 14540 | 12080 |
| **A-6** | 20 | 20 | 20 | 20 | 20 | 20 | 20 |  | **E-6** | 6176 | 10234 | 1138 | 14918 | 1938 | 2296 | 7061 |
| **A-7** | 20 | 20 | 20 | 20 | 20 | 20 | 20 |  | **E-7** | 15066 | 1561 | 7357 | 13707 | 2739 | 1462 | 16663 |
| **A-8** | 20 | 20 | 20 | 20 | 20 | 20 | 20 |  | **E-8** | 11532 | 7509 | 5641 | 9058 | 4998 | 6819 | 8908 |
| **A-9** | 20 | 20 | 20 | 20 | 20 | 20 | 20 |  | **E-9** | 1927 | 105 | 67 | 105 | 111 | 102 | 125 |
| **A-10** | 20 | 20 | 20 | 20 | 20 | 20 | 20 |  | **E-10** | 28659 | 29862 | 26511 | 15010 | 23425 | 22856 | 22024 |
| **B-1** | 1238 | 536 | 177 | 863 | 283 | 1512 | 808 |  | **F-1** | 34951 | 9144 | 11963 | 27599 | 9192 | 17666 | 31054 |
| **B-2** | 582 | 110 | 235 | 298 | 178 | 317 | 291 |  | **F-2** | 21012 | 26074 | 6802 | 13162 | 10169 | 15506 | 15884 |
| **B-3** | 1010 | 165 | 634 | 316 | 122 | 159 | 325 |  | **F-3** | 15205 | 5446 | 8761 | 15108 | 7307 | 20299 | 9092 |
| **B-4** | 143 | 43 | 20 | 166 | 20 | 20 | 111 |  | **F-4** | 23124 | 20963 | 13260 | 20140 | 23473 | 17585 | 28019 |
| **B-5** | 2329 | 3189 | 105 | 2412 | 2014 | 2769 | 7081 |  | **F-5** | 18166 | 10758 | 5923 | 7053 | 6416 | 14461 | 17180 |
| **B-6** | 2164 | 374 | 845 | 2044 | 678 | 2322 | 2534 |  | **F-6** | 6740 | 5049 | 1372 | 6786 | 1974 | 1826 | 3980 |
| **B-7** | 376 | 118 | 196 | 395 | 119 | 122 | 322 |  | **F-7** | 30441 | 27557 | 21141 | 17585 | 24227 | 31597 | 28029 |
| **B-8** | 4678 | 1746 | 1058 | 2451 | 1238 | 1741 | 1801 |  | **F-8** | 17787 | 5563 | 13873 | 14749 | 8913 | 10637 | 28174 |
| **B-9** | 160 | 52 | 20 | 88 | 20 | 20 | 20 |  | **F-9** | 10904 | 782 | 4395 | 5024 | 506 | 2991 | 3530 |
| **B-10** | 229 | 698 | 141 | 146 | 103 | 40 | 116 |  | **F-10** | 32321 | 42746 | 49438 | 31857 | 32876 | 36968 | 44008 |
| **C-1** | 720 | 207 | 544 | 414 | 149 | 408 | 295 |  | **G-1** | 28975 | 39922 | 10545 | 65972 | 33777 | 36192 | 111226 |
| **C-2** | 1810 | 74 | 716 | 1056 | 71 | 1637 | 1770 |  | **G-2** | 38452 | 38152 | 40959 | 87480 | 31212 | 29097 | 29456 |
| **C-3** | 3813 | 857 | 1085 | 3969 | 625 | 2018 | 4137 |  | **G-3** | 44270 | 46027 | 32160 | 58884 | 61243 | 52859 | 63905 |
| **C-4** | 6300 | 1683 | 5225 | 1534 | 1593 | 3100 | 9079 |  | **G-4** | 24343 | 52346 | 42980 | 33249 | 43716 | 63769 | 34374 |
| **C-5** | 2876 | 349 | 1576 | 1500 | 199 | 635 | 2577 |  | **G-5** | 21794 | 19440 | 9368 | 11231 | 12731 | 38088 | 14751 |
| **C-6** | 3231 | 596 | 655 | 2148 | 297 | 734 | 1644 |  | **G-6** | 6947 | 2687 | 2476 | 3622 | 3472 | 2140 | 5465 |
| **C-7** | 4504 | 849 | 3278 | 2946 | 1205 | 3818 | 3532 |  | **G-7** | 28765 | 10728 | 30354 | 29242 | 18709 | 22132 | 24443 |
| **C-8** | 10880 | 8462 | 4837 | 4179 | 5089 | 3688 | 9194 |  | **G-8** | 59177 | 87480 | 66804 | 87480 | 68475 | 50448 | 67669 |
| **C-9** | 1293 | 834 | 347 | 1913 | 479 | 407 | 950 |  | **G-9** | 25399 | 28482 | 16239 | 17919 | 33474 | 52099 | 19447 |
| **C-10** | 6795 | 377 | 2812 | 2636 | 858 | 3267 | 4079 |  | **G-10** | 5439 | 3405 | 4396 | 4125 | 4238 | 7584 | 8009 |
| **D-1** | 4383 | 409 | 1327 | 2775 | 849 | 755 | 4591 |  | **H-1** | 19784 | 41111 | 17317 | 71244 | 29956 | 45747 | 23798 |
| **D-2** | 7783 | 838 | 3820 | 5430 | 1175 | 1437 | 6678 |  | **H-2** | 8520 | 3597 | 9254 | 6511 | 4966 | 14802 | 5758 |
| **D-3** | 12909 | 1906 | 2464 | 9053 | 2645 | 2372 | 11396 |  | **H-3** | 57159 | 1737 | 51149 | 41663 | 2438 | 24601 | 69424 |
| **D-4** | 10772 | 12467 | 3329 | 14433 | 7739 | 9606 | 17925 |  | **H-4** | 24916 | 10856 | 19116 | 14085 | 16571 | 19126 | 23437 |
| **D-5** | 7578 | 295 | 6144 | 1427 | 282 | 695 | 7475 |  | **H-5** | 31829 | 16794 | 19484 | 41826 | 22386 | 24083 | 15974 |
| **D-6** | 13125 | 4858 | 6593 | 16601 | 2725 | 6290 | 18558 |  | **H-6** | 29169 | 13554 | 12986 | 14635 | 13072 | 23169 | 19425 |
| **D-7** | 5256 | 240 | 1408 | 3596 | 280 | 800 | 2281 |  | **H-7** | 19683 | 17564 | 11327 | 25033 | 10581 | 11132 | 28237 |
| **D-8** | 11482 | 19535 | 4888 | 17974 | 11281 | 17793 | 26368 |  | **H-8** | 29448 | 15898 | 26115 | 18254 | 18126 | 30884 | 27772 |
| **D-9** | 13968 | 8578 | 15227 | 10457 | 9136 | 12950 | 8920 |  | **H-9** | 22052 | 10117 | 26701 | 10013 | 16166 | 17345 | 24912 |
| **D-10** | 24903 | 5584 | 9240 | 11609 | 7029 | 9218 | 26762 |  | **H-10** | 186372 | 87480 | 66049 | 87480 | 67036 | 87480 | 87480 |
